# Wearable Cardiorespiratory Monitoring Employing a Multimodal Digital Patch Stethoscope: Estimation of ECG, PEP, LVET and Respiration Using a 55 mm Single-Lead ECG and Phonocardiogram

**DOI:** 10.3390/s20072033

**Published:** 2020-04-04

**Authors:** Michael Klum, Mike Urban, Timo Tigges, Alexandru-Gabriel Pielmus, Aarne Feldheiser, Theresa Schmitt, Reinhold Orglmeister

**Affiliations:** 1 Department of Electronics and Medical Signal Processing, Technische Universität Berlin, Einsteinufer 17, 10587 Berlin, Germany; m.urban@campus.tu-berlin.de (M.U.); timo.tigges@tu-berlin.de (T.T.); a.pielmus@tu-berlin.de (A.-G.P.); theresa.schmitt@campus.tu-berlin.de (T.S.); reinhold.orglmeister@tu-berlin.de (R.O.); 2 Department of Anesthesiology, Intensive Care Medicine and Pain Therapy, Evang. Kliniken Essen-Mitte, Huyssens-Stiftung/Knappschaft, Henricistr. 92, 45136 Essen, Germany; a.feldheiser@kem-med.com; 3 Department of Anesthesiology and Intensive Care Medicine, Charité - Universitätsmedizin Berlin, Campus Virchow-Klinikum, 13353 Berlin, Germany and Charité Campus Mitte, 10117 Berlin, Germany

**Keywords:** ECG, PEP, LVET, respiration rate, wearable cardiorespiratory monitoring, patch, digital stethoscope, ECG-derived respiration, phonocardiogram-derived respiration, neural network

## Abstract

Cardiovascular diseases are the main cause of death worldwide, with sleep disordered breathing being a further aggravating factor. Respiratory illnesses are the third leading cause of death amongst the noncommunicable diseases. The current COVID-19 pandemic, however, also highlights the impact of communicable respiratory syndromes. In the clinical routine, prolonged postanesthetic respiratory instability worsens the patient outcome. Even though early and continuous, long-term cardiorespiratory monitoring has been proposed or even proven to be beneficial in several situations, implementations thereof are sparse. We employed our recently presented, multimodal patch stethoscope to estimate Einthoven electrocardiogram (ECG) Lead I and II from a single 55 mm ECG lead. Using the stethoscope and ECG subsystems, the pre-ejection period (PEP) and left ventricular ejection time (LVET) were estimated. ECG-derived respiration techniques were used in conjunction with a novel, phonocardiogram-derived respiration approach to extract respiratory parameters. Medical-grade references were the SOMNOmedics SOMNO HD^TM^ and Osypka ICON-Core^TM^. In a study including 10 healthy subjects, we analyzed the performances in the supine, lateral, and prone position. Einthoven I and II estimations yielded correlations exceeding 0.97. LVET and PEP estimation errors were 10% and 21%, respectively. Respiratory rates were estimated with mean absolute errors below 1.2 bpm, and the respiratory signal yielded a correlation of 0.66. We conclude that the estimation of ECG, PEP, LVET, and respiratory parameters is feasible using a wearable, multimodal acquisition device and encourage further research in multimodal signal fusion for respiratory signal estimation.

## 1. Introduction

### 1.1. Clinical Background and State-of-the-Art

Of all worldwide deaths, 31.3% were caused by cardiovascular diseases (CVD) in 2016, making them the most common cause of death. Amongst the noncommunicable diseases, respiratory issues are the third leading cause of death worldwide with 6.7% [1]. It is now a well-known fact, however, that respiration-related illnesses can also significantly increase the risk for CVD and other diseases. Especially in the case of obstructive sleep apnea (OSA), close relations to increased CVD such as hypertension [2] and atrial fibrillation [3] have been reported. At the same time, OSA is one of the most common sleep disorders with an estimated 936 million people affected worldwide [4]. Respiratory parameters also play an important role in the clinical, postanesthetic routine. It has been shown that respiratory complications are major causes of prolonged hospital stay, poor overall outcomes, and increased mortality [5,6,7]. Outbreaks of respiratory syndromes such as severe acute respiratory syndrome (SARS), Middle East respiratory syndrome (MERS), and the currently ongoing COVID-19 pandemic [8] further emphasize the importance of tools for an early diagnosis [9]. From the described situation, it becomes evident that cardiorespiratory monitoring is one of the major fields to be addressed in the future.

During the last two decades, rapid developments in battery technology, low-power embedded processors, and integrated sensors have led to technologies being increasingly wearable. Current generations of wearable, noninvasive biosignal acquisition systems can be roughly categorized by their runtime and the physiological parameters of interest [10], as well as their implementation, biosignal selection, and whether or not they are organized into networks [11]. The physiological parameters addressed are oriented on the identified large health issues of the present and future. It is therefore consequent that a large number of systems focus on the cardiovascular system, activity measures, the respiratory system, and the metabolism. The selection of biosignals varies with the use-case and implementation. Commonly found modalities are the ECG, accelerometry, gyrometry, photoplethysmography, galvanic skin response, body temperature, bioimpedance methods, blood glucose, and ambient parameters. Systems can be designed to operate on their own or in networks such as body area networks, body sensor networks, or wireless sensor networks. Due to a variety of materials available ranging from standard printed circuit boards (PCB) and flexible PCBs to textile integrated electronics [12], implementations of wearable biosignal acquisition systems usually range from sensor shirts over chest straps, wrist-bands, and necklaces, to small adhesive patches implemented using flexible materials [13].

The electrocardiogram (ECG) is widely considered as a gold standard for the non-invasive assessment of the cardiovascular system and detection of CVD. The resting ECG is an effective screening method for athletes [14] and a tool for cardiovascular risk assessment in asymptomatic adults [15]. In a chest pain emergency situation, obtaining an ECG within 10 min is recommended [16]. Transient symptoms such as arrhythmias are monitored using 24 h Holter ECG systems or over days or weeks using long-term systems, especially preceding and following ablation therapy [17]. Most of these systems still follow a traditional lead configuration with large inter-electrode spacing. It has been shown, however, that it is possible to synthesize multiple ECG leads from only a few standard measurements [18]. Only a few studies have investigated the possibility to obtain standard leads from short inter-electrode ECG lead systems [19], but some findings suggest that even a 12-channel ECG reconstruction could be possible using a single 5 cm patch device, which incorporates multiple small-distance ECG lead recordings [20]. Even though the lead field theory seems to support the concept of short-distance ECG recordings, no recommendations on application-specific placements have been established yet, and the evaluation of methods beyond ECG lead estimation from short-distance ECG leads is yet sparse [21].

While the ECG reflects the electrical properties of the heart, structural abnormalities or defects do not necessarily manifest themselves in the ECG, but in abnormal heart sounds and murmurs [22]. These changes can be assessed by the phonocardiogram (PCG). While stethoscope auscultation is a valuable and oftentimes first applied tool in primary health care, its digitization is a relatively recent development leading to applications including classification of mitral valve prolapse [23] and the detection of other abnormal heart sounds [24]. Currently available digital stethoscopes are designed close to the traditional implementation [25], with a few exceptions lately emerging [26]. To the best knowledge of the authors, no wearable digital stethoscope implementation was available until very recently.

Combining ECG and PCG signals, systolic time intervals (STI) can be derived. STIs are known to relate closely to ventricular failure, the rate of ventricular pressure rise, preload, and other indices [27]. Especially the pre-ejection period (PEP) and the left ventricular ejection time (LVET) have been found to be closely related to these measures. STIs provide insights into the temporal structure of the mechanical heart activity and can therefore contribute additional vital information about the cardiovascular system. Several approaches to assessing STIs are available, including echocardiography, impedance cardiography (ICG), photoplethysmography, seismocardiography (SCG), and PCG. Considering echocardiography as the reference, the PCG approach outperforms other methods when PEP and LVET are to be estimated [28], and PCG and SCG deliver comparable results estimating total systolic time and the electromechanical delay [29]. Therefore, several approaches of estimating STI from ECG and PCG have been reported [30,31]. Despite the work available on PEP estimation using the PCG, there seem to be diverging opinions about whether or not PCG can be used for PEP estimation in principle [29,32].

Assessing the function of the respiratory system is usually bound to obtrusive sensors. Especially in the context of sleep testing, flow sensors such as face masks or nasal cannula pressure sensors and effort sensors such as respiratory inductance plethysmography chest and abdominal belts are still recommended [33]. While the development of an unobtrusive home sleep test system took important steps forward, the expert opinion remains that further research regarding the minimum number of parameters and methods of signal acquisitions is required [34]. A promising technique for unobtrusive respiratory signal estimation is ECG-derived respiration (EDR), which is commonly used in conjunction with other signal modalities such as photoplethysmography (PPG) [35]. An obvious advantage of EDR in the case of an available ECG is that no additional sensor is needed to estimate respiratory parameters. It is widely accepted that the ECG is modulated in three main aspects by the respiration: baseline wandering, heart rate, and QRS morphology. EDR methods aim to use one or more of these measures to estimate respiratory parameters. The extraction of the heart rate is less sensitive to ECG position and electrode distances due to the electrically strong QRS complex. The usability of morphological features, however, more strongly depends on the lead location. Especially in the context of small sensor systems placed at non-standard positions, this becomes important [36].

Besides ECG and PPG based methods, several additional concepts of estimating respiratory parameters have emerged. Other approaches used to estimate respiratory signals include respiratory sounds, breathing air temperature, humidity and components [37], the oscillometric cuff pressure, Korotkoff sounds, as well as the seismocardiogram (SCG) [38]. For the latter, it has been shown that the morphology of the vibrational cardiac waveforms varies with the respiratory volume [39,40], and methods of extracting respiratory phases [41,42], effort [43] and breathing states such as normal, breathless, long, and labored [44] have been proposed. However, the interindividual morphological variations, as well as dependencies on numerous parameters including subject position, are still challenging [45]. SCG and PCG are closely related [46], with differences mostly defined by their frequency ranges [47]. However, the application of PCG signals for respiratory signal estimation seems not to have been proposed yet. The authors assume that one reason is the unavailability of appropriate long-term monitoring stethoscope systems.

### 1.2. Scope of the Presented Work

**Figure 1:**
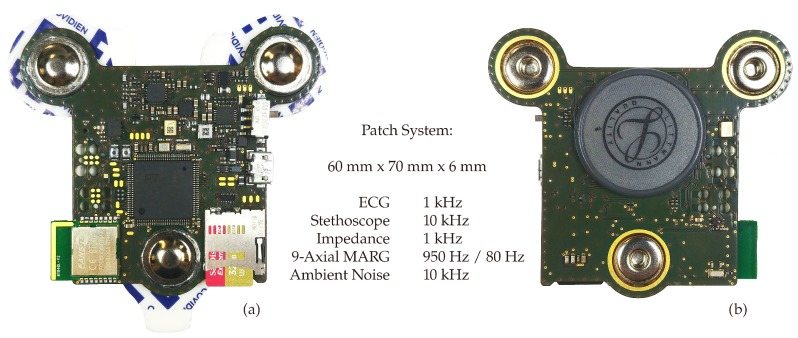
The wearable multimodal stethoscope patch. (**a**) Front view. (**b**) Back view with ECG / IP electrode connectors and 28 mm infant stethoscope membrane.

With strong evidence that continuous, long-term cardiovascular and respiratory monitoring can improve clinical outcome [48], optimize therapy success evaluation [49], and be an effective strategy to assess sleep apnea [50], it is time to introduce a paradigm shift. Unimodal acquisition systems, such as wearable ECGs, can provide vital information. The real strength of wearable monitoring, however, lies beyond: in multimodality matched with unobtrusiveness. We recently presented a wearable, multimodal digital stethoscope patch, shown in Figure 1. The system incorporates single-lead ECG and impedance pneumography at an electrode distance of 55 mm, 9-axial magnetic, angular rate and gravity (MARG) sensors, a digital stethoscope and ambient sound recording in a 60 mm × 70 mm × 6 mm device [51]. In its current implementation, the system employs a thin 450 mAh lithium-polymer battery and realized runtimes exceeding 10 h even though not being optimized for low energy consumption yet.

**Figure 2:**
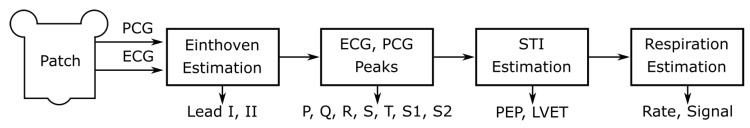
Overview of the processing steps and estimated modalities throughout this work.

In this work, we present three distinct cardiovascular and respiratory monitoring applications using the ECG and digital stethoscope subsystems of our novel, multimodal wearable. We start by evaluating the performance of estimating standard Einthoven leads from the single, 55 mm short-distance lead implemented in the patch. Using the derived standard ECG leads, we classify the ECG fiducial points and the PCG S1 and S2 peaks in the stethoscope signal. This information is used to estimate PEP and LVET. Finally, respiratory signals and rates are estimated using ECG-derived respiration techniques combined with a novel phonocardiogram-derived respiration (PDR) approach. Figure 2 gives a simplified overview of the processing steps. We evaluate our approaches using two commercially available reference systems (SOMNOmedics SOMNO HD^TM^ and Osypka ICON-Core^TM^) acquiring reference ECG signals, PEP, LVET, and respiratory flow in a study including 10 healthy subjects with 33 min measurements each in different body positions. We aim to accelerate the development of truly multimodal, wearable biosignal acquisition and processing towards holistic long-term monitoring concepts.

## 2. Materials and Methods

### 2.1. Data Acquisition

**Figure 3:**
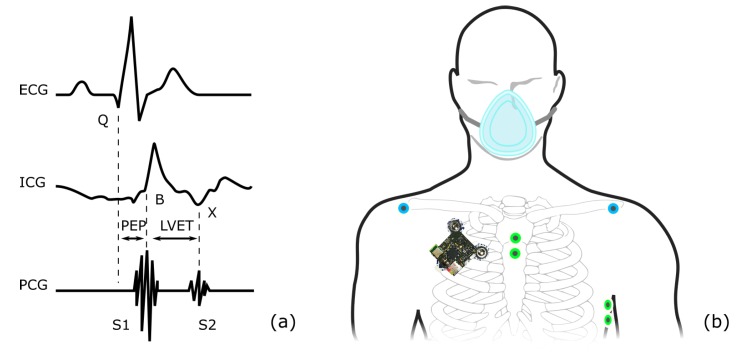
(**a**) PEP and LVET definitions. (**b**) Sensor placement throughout the study.

The data for the presented work were acquired using our previously described wireless, multimodal stethoscope patch shown in Figure 1. Out of the numerous sensors provided by the patch, we used the ECG (1 kHz, 24 bit) and the digital stethoscope (10 kHz, 32 bit) for the applications presented here. Two commercially available biosignal acquisition systems were used alongside the patch to acquire reference data. The Osypka Medical GmbH ICON-Core^TM^ monitor was employed to record a bioimpedance signal (200 Hz), an ECG corresponding to the Einthoven II lead (200 Hz), as well as beat-to-beat reference values for PEP and LVET (resolution of 5 ms). The ICON-Core calculates the PEP as the time between the Q-peak in the ECG and the B-point in the ICG signal. The LVET is calculated as the time between the B-point and the X-point in the ICG. Figure 3a visualizes the PEP and LVET definitions used throughout this work. The SOMNOmedics GmbH SOMNO HD^TM^ polygraphy system was used to record reference respiratory flow (512 Hz) using a pneumotachometer and facial mask, as well as a second ECG corresponding to Einthoven I (1024 Hz). The patch system was attached at an angle of 45° between the first and second intercostal space (ICS) at the right midclavicular line. The four electrodes of the ICON-Core were attached at the first and second ICS on the sternum, as well as at the fifth and sixth ICS on the left midaxillary line. The SOMNO HD ECG electrodes were placed according to the Einthoven I lead, and the facial mask was secured using appropriate rubber material. Figure 3b gives a visual representation of the sensor placement.

We conducted a study including 10 subjects (26 ± 2 years, 2 female), which was approved by the TU Berlin Ethics Committee of the Department of Psychology and Ergonomics under the tracking number KL_01_20180117. Each subject was instructed by a custom app to breathe pre-defined, 11 min long patterns of varying combinations of breathing frequencies (8, 16, 24 breaths per minute (bpm)), breathing depths (shallow, normal), and 30 s simulated apnea (breath holding). This pattern sequence was recorded in the supine, lateral, and prone position, resulting in 33 min of data per subject.

### 2.2. Regression Models and Cross-Validation

**Figure 4:**
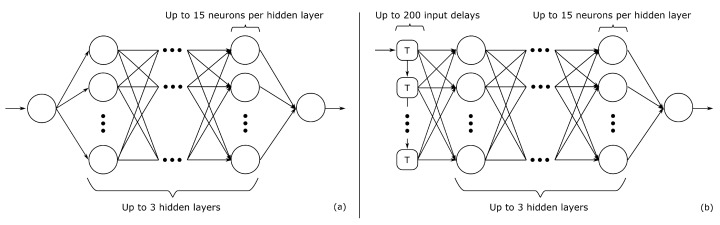
Neural network regression model configurations used throughout this work: (**a**) multi-layer perceptrons. (**b**) time-delay neural networks.

Throughout our work, we used three model classes for regression analysis: polynomials up to the order of three, multi-layer perceptrons (MLP) with two to three hidden layers with two to fifteen neurons each, and time-delay neural networks (TDNN) with the same layer configuration as the MLPs and input delay lines with lags between 1 and 1 to 200 taps. For multiple-input regression problems in the polynomial case, multiple linear regression with polynomial models without cross-terms was employed, following:

(1) $$y=a_{0}+\sum_{m=1}^{M}\sum_{k=1}^{K}a_{mk}x_{k}^{m},$$

where $x_{k}^{m}$ are the *K* independent variables of the power of one to the order of the polynomial *M* and $a_{0}$ is the constant. Figure 4 gives a visual representation of the neural network model configurations, which can be easily expanded for multiple input regression problems.

Prior to training, training and testing observation and target data were normalized to zero mean and unit variance. The mean and variance of the training target data were saved along with the trained model as an estimate of the target distribution. In the test phase, the estimated target data were denormalized using the distribution estimated in the training phase.

**Figure 5:**
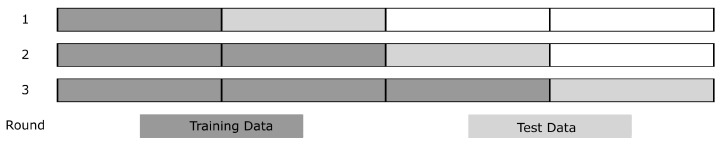
Example of the data split in forward-chaining cross-validation with nCV=3 validation rounds.

All reported regression performances were obtained using an nCV-times nested forward chaining cross-validation scheme. Figure 5 gives an Example for nCV=3. Data were divided into nCV+1 continuous sections without randomization. In the first validation round, the first section was used for training and the second section for testing. In the next validation round, the training data consisted of all the previously used training and testing sections, thus Section 1 and Section 2. The test dataset consisted of the next section, thus the third. This scheme was followed until all data were used. The mean and standard deviation of the performance distribution are reported.

### 2.3. Performance Metrics

We used several metrics throughout our work to characterize the performance of estimations Est relative to reference values Ref. The systematic error, or bias, was characterized using the mean error,

(2) $$ME=\frac{1}{N}\sum_{i=1}^{N}Est\left(i\right)-Ref\left(i\right),$$

while absolute errors were evaluated by the mean absolute error:

(3) $$MAE=\frac{1}{N}\sum_{i=1}^{N}\left|Est\left(i\right)-Ref\left(i\right)\right|.$$

In order to provide more population independent measures, we provided two relative, percentage metrics chosen based on the type of data. For strictly non-zero data, we employed the mean absolute percentage error:

(4) $$MAPE=\frac{1}{N}\sum_{i=1}^{N}|\frac{Est(i)-Ref(i)}{Ref(i)}|*100\;\%.$$

For data including or approaching zero, such as ECG or respiratory signals, the normalized mean squared error:

(5) $$NMSE=\frac{\sum_{i=1}^{N}{\left(Est\left(i\right)-Ref\left(i\right)\right)}^{2}}{\sum_{i=1}^{N}Ref{\left(i\right)}^{2}}*100\;\%$$

was employed to avoid singularity effects. In order to assess correlations, we used the Pearson linear correlation *r*. Model selection was performed using the Bayesian information criterion:

(6) $$BIC=N*ln\left(\frac{SSE}{N}\right)+ln\left(N\right)*K,$$

where SSE is the sum of squared errors, *N* is the number of observations, and *K* is the model order.

### 2.4. Einthoven Lead Estimation

Prior to the ECG lead estimation using the patch ECG, all three ECG signals were resampled to 200 Hz and low-pass filtered at a corner frequency of 75 Hz using a zero-phase FIR filter with an order of 400. Their baselines were removed by subtracting 0.5 Hz moving-average filtered signals. Finally, a 50 Hz notch filter with a quality factor of q=10 was employed to reject power line interference.

Using the three models and cross-validation schemes discussed, the patch ECG signals were fitted to the SOMNO HD ECG signal to estimate Einthoven Lead I and to the ICON-Core ECG signal to estimate Einthoven Lead II. Models were fitted for each proband and each position individually, and the selection of the model parameters was based on the minimum of the BIC of the validation data, as given in Equation (6).

### 2.5. PEP and LVET Estimation

Prior to the PEP and LVET estimation using the patch ECG and patch stethoscope signals, the patch ECG signals were processed as described in Section 2.4. The stethoscope signals were band-pass filtered at corner frequencies of 5 Hz and 250 Hz using a zero-phase FIR filter with an order of 400 and resampled to 5 kHz.

**Figure 6:**
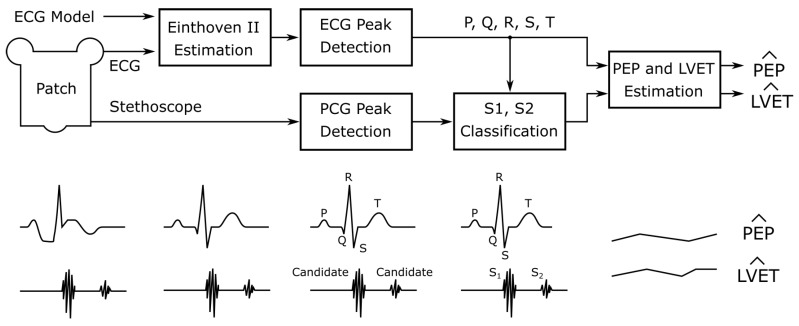
ECG and PCG peak detection and classification of S1 and S2 candidates before PEP and LVET estimation.

Due to the definition of the PEP, the non-standard ECG lead recorded by the patch was suboptimal for its estimation. It was therefore transformed into the Einthoven II lead using the model derived in Section 2.4. The ECG R-peak was then detected using the Pan–Tompkins ECG peak detection algorithm [52]. From the R-peak, P, Q, S, and T were identified using successive rule based windowing and local maximum and minimum detection. The start, end, and peak of S1 and S2 within the stethoscope PCG signals were detected using a modified approach of the empirical wavelet transform and the instantaneous phase based method proposed by Varghees et al. [53], combined with an ECG based PCG peak classification step. Due to the placement of the patch, the sounds differed from standard heart auscultation and were contaminated by respiratory sounds. Figure 6 gives a structural overview of the procedure.

Within the original algorithm, we altered the thresholding phase from the original, signal-dependent, but fixed threshold to a dynamic threshold calculated in 10 s windows using the standard deviation. In order to robustify the PCG peak detection in the presence of noise, several additional processing steps followed the raw detection phase detailed by Varghees et al., replacing the original S1 and S2 classification approach. First, identified peak candidates were analyzed for completeness. For each peak candidate, a start, local maximum, and end had to be available. If one or more were missing, the candidate was discarded. Using the ECG and the current heart rate, a window from each R-peak to 75% of the current heart cycle length was defined where S1 and S2 candidates were identified. If exactly two peak candidates were found in that window, the peak closer to the R-peak was defined as S1 and the second one as S2. If less than two peaks were found, the peak was discarded. When more than two candidates were found in the window, the peak closest to the R-peak was defined as S1, and the peak closest to the T-wave was defined as S2. After peak detection and classification, the PEP was calculated from the ECG and stethoscope as:

(7) $$PEP\left(n\right)=t_{S1_{peak}}\left(n\right)-t_{Q}\left(n\right),$$

and the LVET as:

(8) $$LVET\left(n\right)=t_{S2_{peak}}\left(n\right)-t_{S1_{peak}}\left(n\right),$$

where $t_{S1_{peak}}\left(n\right)$, $t_{Q}\left(n\right)$, and $t_{S2_{peak}}\left(n\right)$ are vectors containing the temporal location in seconds of the respective elements for each heartbeat *n*. S1_peak_ and S2_peak_ are the peaks of the heart sounds S1 and S2, respectively, and Q is the Q-wave of the QRS complex of the ECG.

### 2.6. Electrocardiogram- and Phonocardiogram-Derived Respiration

We derived respiratory surrogates from both the ECG, using ECG-derived respiration (EDR) techniques, and the PCG, using our proposed novel method of PCG-derived respiration (PDR). We used the ECG and PCG signals, as well as their previously described fiducial points. Table 1 gives an overview of the twelve employed features.

The EDR and PDR features can be divided into four classes: timing based (HR, LVET, PEP), area based ($QRS_{area}$, $S1_{area}$, $S2_{area}$), amplitude based ($QRS_{amp}$, $S1_{amp}$, $S2_{amp}$), and morphology based ($QRS_{PCA}$, $S1_{PCA}$, $S2_{PCA}$). The definitions of PEP and LVET are given in Equations (7) and (8). The heart rate (HR) in beats per minute at the current beat *n* was calculated from the location of the R-peaks of the ECG as:

(9) $$HR\left(n\right)=60\frac{s}{min}*\frac{1}{t_{R}\left(n\right)-t_{R}\left(n-1\right)},$$

where $t_{R}\left(n\right)$ is the time of the R-peak in beats *n* in seconds. The area based features were calculated in windows of length $2*QRS_{wind}$, $2*S1_{wind}$, and $2*S2_{wind}$ around the ECG, S1, and S2 peaks, respectively. The QRS window length was set to 250 ms. The S1 and S2 window lengths were the average S1 and S2 segment lengths for each analyzed subject. The beat-to-beat areas were then calculated as:

(10) $$QRS_{area}\left(n\right)=\int_{t_{QRS_{peak}}\left(n\right)-QRS_{wind}}^{t_{QRS_{peak}}\left(n\right)+QRS_{wind}}\left|ECG(t)\right|dt,$$

(11) $$S1_{area}\left(n\right)=\int_{t_{S1_{peak}}\left(n\right)-S1_{wind}}^{t_{S1_{peak}}\left(n\right)+S1_{wind}}\left|PCG(t)\right|dt,$$

and:

(12) $$S2_{area}\left(n\right)=\int_{t_{S2_{peak}}\left(n\right)-S2_{wind}}^{t_{S2_{peak}}\left(n\right)+S2_{wind}}\left|PCG(t)\right|dt.$$

The PCG amplitude features $S1_{amp}$ and $S2_{amp}$ were calculated as the difference between the maximum and the minimum in the respective peak’s window. The ECG amplitude feature $QRS_{amp}$ was defined as the R-to-S amplitude.

For the morphology based features, the linear principal component analysis (PCA) was calculated over the previously defined windows of the respective signals. In the ECG case, the Eigenvector with the largest Eigenvalue strongly corresponded to the respiration and could therefore be used as a morphology based feature [54]. We adopted this approach for the S1 and S2 peaks in addition to the QRS complex.

**Figure 7:**
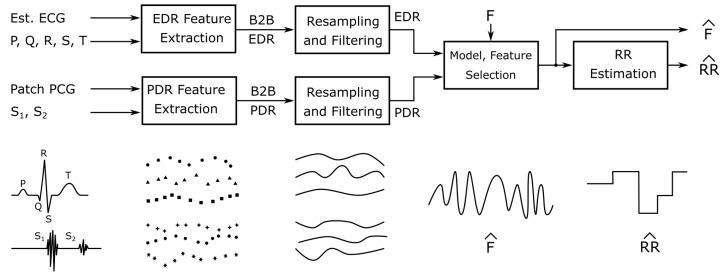
Beat-to-beat (B2B) ECG and PCG derived respiration for estimating the respiratory flow $\hat{F}$ and rate $\hat{RR}$ using the estimated ECG and the PCG signals in a model and feature selection framework.

The resulting twelve beat-to-beat feature vectors were then up-sampled to 20 Hz using linear interpolation. The first derivative of the up-sampled signals was low-pass filtered using a zero-phase FIR filter with an order of 200 at a corner frequency of 1 Hz. A 0.05 Hz baseline was removed. Figure 7 gives an overview of the processing steps.

The resulting twelve EDR and PDR feature vectors were processed in a nested cross-validation model and feature selection framework similar to that discussed in [55]. Using the models presented in Section 2.2, features were selected by a forward wrapping approach. For each model-feature combination, cross-validated performances of the estimated flow signal $\hat{F}$ and respiratory rate $\hat{RR}$ relative to the SOMNO HD reference respiratory flow *F* and rate RR were calculated. Based on the linear correlation between *F* and $\hat{F}$ and the respiratory rate estimation error, the optimal model-feature combinations were found. Respiratory rates were estimated in 30 s windows with 50% overlap using the maximum of the FFT between 4 and 40 bpm.

## 3. Results

### 3.1. Einthoven Lead Estimation

**Figure 8:**
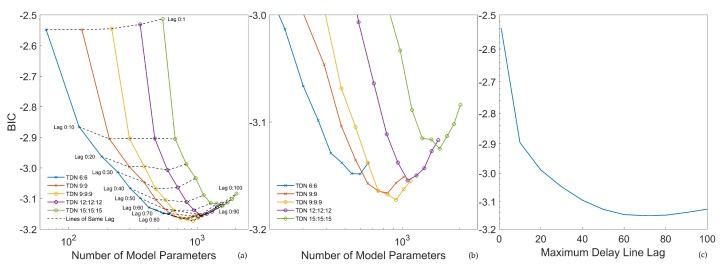
BIC model selection for Einthoven ECG lead estimation, TDNNs only: (**a**) Selection of models and lines of constant lag length. (**b**) Minimum BIC values. (**c**) BIC over the number of delay line lags.

The results of the model selection step for the ECG lead estimation are visualized in Figure 8a–c. As both polynomial models, as well as MLPs performed significantly worse than TDNNs, only a selection of the results of the TDNNs is shown. The optimal polynomial model was of the order of nine. The optimal MLP model consisted of three layers with twelve neurons each. In (a), dashed lines represent models with the same lag parameters. It became apparent that increasing the maximum lag significantly decreased the BIC. Increasing the model complexity in the hidden layers while keeping the lag parameters constant had a smaller impact on the BIC. In (c), the average BIC over maximum model lag is given. It became evident that a lag value of 70 to 80 was optimal. In (b), the lowest points of the BIC curves are given. Based on that criterion, the TDNN with three hidden layers and nine neurons each with a delay line of zero to 80 lags was the optimal model and was therefore chosen for subsequent ECG analysis.

The average ECG estimation performances over all subjects and positions are given in Table 2 for the optimal model of each of the three model classes. In general, MLPs increased the performance only slightly compared to polynomial models. Using TDNNs however, the performance increased significantly. With the optimal TDNN model, the estimation of the second Einthoven lead showed a linear correlation of 0.99 and a relative error of 5.5%. A slightly lower correlation was achieved for the first Einthoven lead, which showed a linear correlation of 0.97 and a relative error of 1.6%. No significant performance differences between the supine, lateral, and prone position were observed.

**Figure 9:**
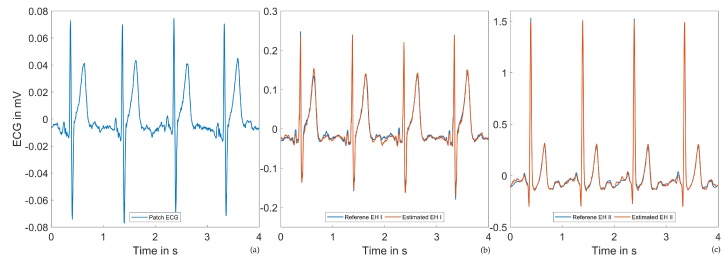
Example ECG signals of Subject 1 in the supine position: (**a**) Patch ECG. (**b**) Reference and estimated Einthoven I ECGs. (**c**) Reference and estimated Einthoven II ECGs.

**Figure 10:**
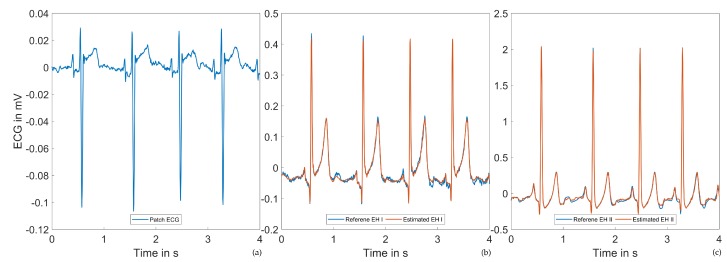
Example ECG signals of Subject 2 in the supine position: (**a**) Patch ECG. A high intersubject variability of the patch ECG lead can be observed when compared to Figure 9a. (**b**) Reference and estimated Einthoven I ECGs. (**c**) Reference and estimated Einthoven II ECGs.

Figure 9 and Figure 10 show ECG excerpts of two different subjects in the supine position at the same time within the measurement protocol. In (a), the 55 mm patch ECG is shown; (b) and (c) show the reference Einthoven leads, as well as the estimated ECG signals. While the Einthoven leads were comparable between the two subjects, the patch ECG leads differed considerably. Throughout our investigations, we observed high intersubject variability of the patch ECG lead morphology. Despite these variations, the Einthoven leads could always be estimated with the given performance.

### 3.2. PEP and LVET Estimation

**Figure 11:**
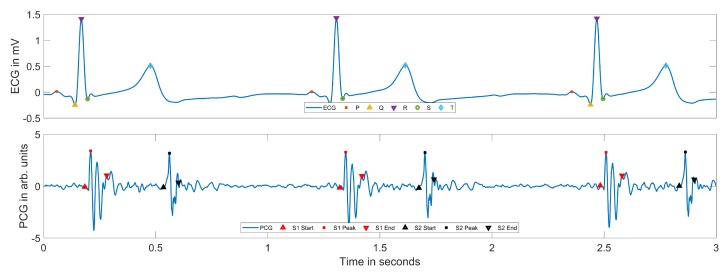
ECG and PCG signals with detected fiducial points.

Figure 11 exemplarily shows an ECG and PCG signal excerpt with the detected fiducial points within a simulated apnea phase. The ECG peak detection performed well over all signals due to the high signal quality. In the PCG, the starts and peaks for both S1 and S2 were detected consistently. The duration of the S1 and S2 peaks tended to be under-estimated slightly, due to relatively early S1 end and S2 end detection. In low signal quality scenarios, especially in deeper breathing phases, the PCG peak detection performance was reduced due to the additional noise introduced by the lung sounds. The most common problem was the detection of false peaks, which was reduced by the additional ECG information in the classification phase. In 85.8% of the total stethoscope data, two PCG peaks were identified using the proposed combined ECG and PCG peak detection approach. The lateral position performed best with 89.8%, followed by the supine position with 81.9%. In the prone position, 75.8% of the data were usable for PCG peak detection.

In Table 3, the results of the PEP and LVET estimations for the lateral position are given, as it resembled the typical echocardiography positioning for which published performances were available. In this position, PEP was estimated with an error of 16.1%. The relative LVET estimation error was 7.0%. For long-term ambulatory estimations, however, other positions were of interest as well. We found that the performance of the LVET and PEP estimations differed between different positions. Over all positions and subjects, the PEP was estimated with an ME of 0.4 ms and an MAE of 25.1 ms, which translated to 21.3% relative error. The LVET was estimated with an ME of −3.6 ms and an MAE of 30.5 ms, which was a relative error of 10.0%.

**Figure 12:**
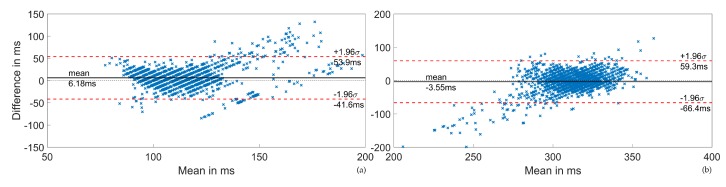
Bland–Altman plots of the STI estimates. (**a**) PEP. (**b**) LVET.

Figure 12 shows Bland–Altman plots of the STI estimates for PEP (a) and LVET (b). The mean reference PEP was 111.4 ms, and the mean reference LVET was 303.4 ms. The visible quantization was a result of the 5 ms resolution of the PEP and LVET reference values from the ICON-Core. The PEP plot shows a series of positive outliers above 150 ms, while in the LVET plot, outliers are more commonly negative and located below 275 ms. Otherwise, no strong trend became evident in the error.

### 3.3. Electrocardiogram- and Phonocardiogram-Derived Respiration

Optimizing the model and feature selection based on the correlation of the estimated flow signal $r_{flow}$, a TDNN with two hidden layers, two neurons each, and a tap delay vector of zero to nine was chosen. Out of the twelve available EDR and PDR features, $HR,QRS_{area},S1_{PCA}$, and $QRS_{amp}$ were selected. Optimizing for a minimal MAE of the respiratory rate estimation $MAE_{bpm}$, a two-layer TDNN with four neurons each and a tap delay line with lags of zero to 14 performed best, and the features $HR,QRS_{amp},S1_{amp},LVET$, and $QRS_{PCA}$ were selected. In Table 4 and Table 5, the performances of the respiratory signal and rate estimations relative to the flow reference are given. Data are shown for the two optimization criteria and individually for all positions with outliers removed. A data point was deemed an outlier if its distance to the median was larger than three scaled median absolute deviations.

In both optimization scenarios, the RR estimation bias was close to zero. The RR estimation error was slightly lower for the corresponding optimized feature selection. The flow signal correlation was higher for the correlation optimized feature set. A consistent finding was the position-dependent performance of all parameter estimations with the supine position performing best, followed by the lateral and the prone position. The relative error more than doubled between the supine and prone position, and the flow correlation was reduced from 0.75 to 0.51. In addition, the number of outliers was highly dependent on the probands’ position. Again, in the supine position, the least amount of outliers were detected. Without outlier removal, the average performance over all postures for the RR estimation was ME=−0.40±3.57 bpm, MAE=1.20±3.38 bpm, $r_{RR}=0.87\pm 0.08$, and MAPE=8.15±23.55%. The best performing individual feature without outlier removal was the HR with a flow correlation of 0.55±0.10, an RR ME of −0.45±1.62 bpm, an RR MAE of 1.88±1.16 bpm, an RR MAPE of 13.21 ± 27.4%, and an RR correlation of 0.82±0.14.

**Figure 13:**
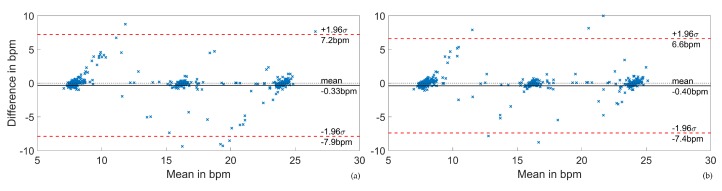
Performance plots of the EDR and PDR based RR estimations over all positions including outliers. (**a**) Correlation optimization. (**b**) Respiration rate optimization.

Figure 13a,b shows Bland–Altman plots of the respiratory rate estimations including all positions for the two optimization targets and without outlier removal. The three distinct respiratory frequencies of the study protocol became apparent. The lower and higher respiratory frequencies were more prone to outliers, while no directly rate-dependent error dependency was found.

**Figure 14:**
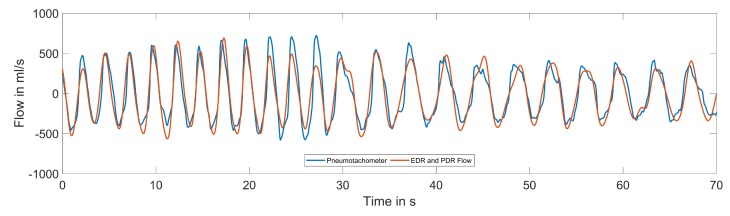
Example reference and estimated respiratory signal using ECG- and PCG-derived respiration.

Figure 14 gives an example of a reference and estimated respiratory flow in two different breathing phases. The estimated breathing signal followed the frequency change and also adapted to the varying breathing depths over time. The more detailed, transient behavior was captured less effectively due to the beat-to-beat nature of the underlying feature basis.

## 4. Discussion

Estimating Einthoven I and II ECG leads from a single 55 mm patch ECG yielded errors below 6% with correlation coefficients above 0.97. This finding confirmed the assumption for Einthoven I and II made by Lee et al. that an ECG patch system of that size could be used to estimate standard ECG leads [20]. By using only a single lead instead of multiple neighboring leads, an important additional restriction was introduced in our work, which further reduced the system complexity. However, in our investigations, linear and MLP models did perform similar to each other and did not reach the performance reported by Lee et al. By introducing TDNNs, similar performance values were achieved. We assume that by using multiple leads oriented differently on the body surface in their regression models, Lee et al. effectively added additional information that was not available in our single-lead approach. However, we can report that for Einthoven I and II, this missing information could be extracted from a single lead, thus removing the need for additional leads. Reviewing patient-specific data, a large morphological inter-subject variability of the minimal ECG lead was found, even though great care was taken applying the patch system in the same position for every subject. We must therefore assume that it is best practice to learn subject-specific regression models and therefore recommend an individualized approach. Future research could analyze the possibility of finding models able to generalize over subjects or cohorts.

The estimation of PEP and LVET from the wearable stethoscope and transformed ECG yielded errors of 10% for LVET and 21% for PEP over all subject positions. In the lateral positions, errors were 7% and 16%, respectively, highlighting the high position dependency of the estimation. The positive PEP and negative LVET outliers reported could be explained by a suboptimal S1 peak detection. The position of the S1 heart sound had a strict lower bound in its temporal position within the heart cycle. It could not be observed before the Q-point of the ECG. In the presence of noise, the distribution of the S1 peak position detection error had therefore a positive bias. Thus, the S1 peak position tended to be over-estimated. Given Equations (7) and (8), it then became plausible that PEP would exhibit more positive and LVET more negative outliers. Compared to the same position and for healthy subjects, the performances reported by Paiva et al. were in the same range, whilst slightly higher [30]. Differences in the performance were most likely due to the automated S1 and S2 annotation process, which suffered from the suboptimal placement of the stethoscope system for the task of STI estimation due to lung sounds. While Paiva et al. placed the stethoscope at the left sternal border, the placement of our patch was chosen according to a wider use-case spectrum and an emphasis on adherence in an ambulatory setting, which resulted in a much larger lung sound content and thus lower overall signal quality. It is worth noting though that similar placement issues are foreseen in a wearable, multimodal sensor application, and therefore, the procedure simulated typical use-cases and performances more accurately. Future research could further analyze the highly subject position-dependent estimation performances and compare SCG and PCG based STI estimation in a wearable context.

The respiratory rate was estimated by the ECG- and PCG-derived respiration algorithm with a mean error of below −0.4 bpm and a mean absolute error below 1.2 bpm. The results were well within the range of other wearable respiration estimation systems using the ECG and an additional sensor, such as accelerometry [56]. The flow signal was estimated with a linear correlation of 0.66 relative to the flow reference. Using the first Eigenvector of the QRS complex, Langley et al. reported correlations to a flow signal of 0.58 [54]. Both the respiration rate and the flow estimation performances were dependent on the subject position, while still giving acceptable results in all three examined postures. It is worth noting that the number of outliers differed significantly between the three subject positions and mainly contributed to the, otherwise small, deviations of the error distributions. Introducing the additional ECG and PCG features compared to the single best ECG feature decreased the relative respiration estimation error from 13% to 8% and increased the flow correlation from 0.55 to 0.66. With an absolute respiration rate error below 1.2 bpm, the estimation of the respiratory signal itself, i.e., flow and tidal volume, should be emphasized in future research to remove the burden of masks, belts, and nasal sensors in respiration measurements.

A limitation of the results discussed is the number of subjects included in the study. A strength, on the other hand, is that each subject was analyzed in the supine, lateral, and prone position for over 11 min each, yielding more than 5 h of total data and more than 20,000 beats automatically analyzed. Due to the different subject positions, a more detailed analysis of the ambulatory use-case was possible. In addition, a range of respiratory frequencies and depths, as well as simulated apneas were part of the protocol. For the STI estimation, the reference system used an ECG-ICG approach, which is known to be less accurate than echocardiography [29], and in the presented case had a resolution of only 5 ms. Despite these limitations, the reference data stemmed from a clinically validated and approved medical device and should therefore be adequately reliable.

## 5. Conclusions

The application of our wearable, multimodal digital patch stethoscope for ECG, PEP, LVET, as well as respiratory rate and flow estimation was presented. It was shown that all three applications could be covered using a 55 mm single lead ECG integrated into a patch stethoscope system in the supine, lateral, and prone positions. In the case of Einthoven I and II, as well as the respiratory rate, the estimations showed very good agreement with the reference systems. PEP and LVET estimation performances were lower, but within the range of published results of other research groups. The slightly lower performances most likely resulted from the suboptimal placement of the stethoscope for cardiac auscultation. From a signal processing point of view, the presented results leave room for further improvements. Clinically, however, the correlations for LVET, PEP, and respiratory flow could be considered as very promising as the good results are substantially beneficial to future patients due to the positive trade-off of being non-invasive and not interfering with the respiration of the patients. Except for the ECG estimation, all parameters exhibited strong subject position-dependent performances. We found strong morphologic intersubject variability in all signals.

We concluded that the estimation of ECG, PEP, LVET, and respiratory parameters is possible based on highly localized, multimodal acquisition systems including an ECG and a stethoscope. However, we recommend deriving subject-specific regression models until more studies are available on generalized intersubject or cohort regression models. Additional research should include the subject position dependency of estimation quality. In order to assess the long-term applicability of the presented system and methods, an evaluation by means of a full polysomnography in parallel with our patch device will be needed. Finally, respiratory signal, flow, and tidal volume estimation appear to be the next logical step after respiratory rate estimation to be able to assess the respiratory apparatus without masks, belts, or nasal sensors. We hope to further motivate and accelerate the development of truly multimodal acquisition systems that would enable the holistic long-term monitoring approach needed to tackle the cardiovascular and respiratory challenges of the future.

## Figures and Tables

**Table 1:** ECG- and PCG-derived respiration features.

Feature Class	Source Signals	Feature Symbol	Description
Timing	ECG	HR	Heart rate
Timing	PCG	LVET	Left ventricular ejection time
Timing	ECG, PCG	PEP	Pre-ejection period
Area	ECG	$QRS_{area}$	QRS complex area
Area	PCG	$S1_{area}$	S1 area
Area	PCG	$S2_{area}$	S2 area
Amplitude	ECG	$QRS_{amp}$	QRS complex amplitude
Amplitude	PCG	$S1_{amp}$	S1 amplitude
Amplitude	PCG	$S2_{amp}$	S2 amplitude
Morphology	ECG	$QRS_{PCA}$	Morphological variations of the QRS complex
Morphology	PCG	$S1_{PCA}$	Morphological variations of the S1 peak
Morphology	PCG	$S2_{PCA}$	Morphological variations of the S2 peak

**Table 2:** Performances of the Einthoven I and II ECG lead estimations from the patch ECG for the optimal polynomial, MLP, and TDNn models.

Target ECG Lead	Model	ME_μV_	MAE_μV_	r	NMSE_%_
Einthoven I	Poly	0.00±0.58	30.6±10.7	0.67±0.18	52±24
Einthoven I	MLP	0.09±0.09	29.5±10.4	0.69±0.17	50±22
**Einthoven I**	**TDNN**	0.11±0.51	9.60±3.10	0.97±0.04	5.5±7.0
Einthoven II	Poly	0.20±2.70	114±49.5	0.75±0.15	42±21
Einthoven II	MLP	0.28±3.5	110±37	0.77±0.14	40±20
**Einthoven II**	**TDNN**	0.70±1.20	23.80±5.7	0.99±0.00	1.6±0.6

**Table 3:** PEP and LVET estimation results for aortic area auscultation and ECG signals in the lateral position.

Position	STI	ME_ms_	MAE_ms_	MAPE_%_
Lateral	PEP	6.18±24.36	17.61±17.92	16.06±16.16
	LVET	−3.55±32.07	21.81±23.78	7.01±7.64

**Table 4:** Performances of the respiratory signal and rate estimations in the individual positions for maximum $r_{flow}$ feature selection optimization. The percentage of outliers removed is given.

Position	r_flow_	ME_bpm_	MAE_bpm_	r_RR_	MAPE_%_	Outliers_%_
**supine**	0.75±0.09	−0.01±0.23	0.18±0.15	0.91±0.07	1.46±1.43	9.60
lateral	0.66±0.11	−0.05±0.30	0.23±0.20	0.85±0.03	1.89±1.93	15.34
prone	0.51±0.10	0.04±0.50	0.35±0.36	0.76±0.11	3.30±3.91	22.73
all	0.66±0.14	−0.03±0.29	0.22±0.19	0.85±0.09	1.86±1.95	16.60

**Table 5:** Performances of the respiratory signal and rate estimations in the individual positions for minimum $MAE_{bpm}$ feature selection optimization. The percentage of outliers removed is given.

Position	r_flow_	ME_bpm_	MAE_bpm_	r_RR_	MAPE_%_	Outliers_%_
**supine**	0.71±0.10	0.01±0.26	0.20±0.16	0.92±0.06	1.64±1.57	9.60
lateral	0.62±0.13	−0.05±0.29	0.21±0.20	0.87±0.06	1.83±1.96	14.20
prone	0.48±0.11	0.00±0.45	0.34±0.29	0.79±0.05	3.14±3.21	20.45
all	0.62±0.14	−0.02±0.33	0.25±0.22	0.87±0.08	2.13±2.25	12.06

## Abbreviations

The following abbreviations are used in this manuscript:

B2B	Beat-to-beat
BIC	Bayesian information criterion
bpm	Breaths per minute
CVD	Cardiovascular disease
ECG	Electrocardiogram
EDR	Electrocardiogram-derived respiration
FFT	Fast Fourier transform
ICG	Impedance cardiography
ICS	Intercostal space
LVET	Left ventricular ejection time
MDPI	Multidisciplinary Digital Publishing Institute
MAE	Mean absolute error
MAPE	Mean absolute percentage error
MARG	Magnetic, angular rate, and gravity
ME	Mean error
MERS	Middle East respiratory syndrome
MLP	Multi-layer perceptron
NMSE	Normalized mean squared error
PCA	Principal component analysis
PCB	Printed circuit boards
PCG	Phonocardiogram
PDR	Phonocardiogram-derived respiration
PEP	Pre-ejection period
PPG	Photoplethysmogram
RR	Respiratory rate
SARS	Severe acute respiratory syndrome
SCG	Seismocardiogram
SSE	Sum of squared errors
STI	Systolic time intervals
TDNN	Time-delay neural network
